# Alcohol increases treatment failure for *Helicobacter pylori* eradication in Asian populations

**DOI:** 10.1186/s12876-023-03002-z

**Published:** 2023-10-25

**Authors:** Jing Yu, Yiming Lv, Peng Yang, Yizhou Jiang, Xiangrong Qin, Xiaoyong Wang

**Affiliations:** 1 Department of Gastroenterology, The Affiliated Changzhou No. 2 People’s Hospital of Nanjing Medical University, 29 Xinglong Lane, Tianning District, Changzhou, 213000 Jiangsu Province China; 2 Graduate School, Dalian Medical University, 9 West Section of Lushun South Road, Lvshunkou District, Dalian, 116000 Liaoning Province China

**Keywords:** *Helicobacter pylori* eradication, Alcohol consumption, Asian populations, Vonoprazan

## Abstract

**Background and Aim:**

Whether alcohol intake is associated with *Helicobacter pylori* (*H. pylori*) eradication failure remains controversial, and this meta-analysis was aimed at investigating the effect of alcohol on the risk of *H. pylori* eradication failure.

**Methods:**

Relevant studies were systematically screened for and retrieved from PubMed and Web of Science (updated to January 2022), and relevant references were manually reviewed. The odds ratios (ORs) and 95% confidence intervals (CIs) were calculated. Subgroup, publication bias, and sensitivity analyses were also conducted.

**Results:**

A total of 40 studies were included in the meta-analysis. No significant association was found between alcohol consumption and the risk of *H. pylori* eradication failure (OR = 1.09, 95% CI, 0.94–1.26). However, in subgroup analyses stratified by region, a positive association was found in Asian patients (OR = 1.23, 95% CI, 1.03–1.47). In Asian patients, alcohol consumption was associated with the risk of *H. pylori* eradication failure when the duration of therapy was > 7 days (OR = 1.17, 95% CI, 1.10–1.25), when the treatment regimen included nitroimidazoles (OR = 1.16, 95% CI, 1.09–1.24), and when patients were treated with bismuth-containing quadruple therapy (OR = 1.17, 95% CI, 1.10–1.25). Alcohol intake > 40 g/day was associated with *H. pylori* eradication failure (OR = 3.17, 95% CI, 1.56–6.41). Moreover, in Asian patients who were administered a vonoprazan (VPZ)-based therapy regimen, alcohol consumption had no effect on *H. pylori* eradication rates (OR = 1.73, 95% CI, 0.98–3.05).

**Conclusion:**

Our meta-analysis clearly showed that a higher daily alcohol intake was associated with a higher risk of *H. pylori* eradication failure in Asian populations. Moreover, a VPZ-based treatment regimen can prevent this effect.

## Introduction

*Helicobacter pylori (H. pylori)* infection is among the most prevalent infections in the world and affects approximately half of the population in Asia [1]. *H. pylori* can cause chronic gastritis, peptic ulcers, stomach cancer, and other digestive system diseases. Treatment of *H. pylori* infection can effectively control the occurrence and development of these diseases [2, 3]. The ideal regimen for treating *H. pylori* infection should return eradication rates of more than 90%–95% and 80% in per-protocol and intention-to-treat analyses, respectively, and should be well tolerated with few adverse effects (< 5%) [4]. However, sequential, concomitant, and bismuth-containing therapies used for *H. pylori* infection in recent years have not achieved satisfactory eradication rates [5]. As the chances of successful eradication decrease with increasing number of follow-up eradication attempts, factors leading to eradication failure should be identified and avoided to the extent possible [6].

Some studies have examined the influence of several factors on *H. pylori* eradication. The most common factors showing significant correlation with *H. pylori* eradication were antibiotic resistance [7, 8], smoking [9], cytochrome P450 (CYP) 2C19 genotype [10], body mass index (BMI) [3, 11], and poor treatment adherence [1, 12]. However, the effect of alcohol on *H. pylori* eradication has been less explored with controversial findings. Alcohol consumption was reported to significantly lower the *H. pylori* eradication rate [11, 12], suggesting its role in the increased risk of *H. pylori* eradication failure. Conversely, some studies have reported no association between alcohol consumption and the risk of *H. pylori* eradication failure [13–15]. Meanwhile, vonoprazan (VPZ), a novel potassium-competitive acid blocker (P-CAB), has been recently approved in Japan for *H. pylori* eradication [16]. Studies have shown that alcohol consumption does not affect the eradication rate of VPZ-based treatment regimens [2, 16, 17]. Therefore, a meta-analysis was conducted to clarify these discrepancies and investigate the association between alcohol consumption and the risk of *H. pylori* eradication failure.

## Methods

This meta-analysis was conducted as per the Preferred Reporting Items for Systematic Reviews and Meta-Analyses (PRISMA-P) guidelines [18]. The research problem was based on the PICO model: P [population]: Studies that recruited patients with *H. pylori* infection as participants; I [intervention or exposure]: alcohol consumption; C [comparison agent]: comparison between the alcohol group and the control group; and O [result]: *H. pylori* eradication.

### Search strategy

A literature search was conducted on PubMed and Web of Science for all relevant literature published before January 2022 using the keywords (*Helicobacter pylori* eradication OR *H. pylori* eradication) AND (alcohol OR drinking). Some relevant studies were included on the basis of a manual review of references as well.

#### Inclusion criteria

In our meta-analysis, we included (1) studies exploring the effect of different levels of alcohol exposure (high *vs*. low; any *vs*. none) on *H. pylori* eradication; (2) studies with data for odds ratios (ORs) available or those in which the number of eradication cases could be obtained according to the drinking category; (3) studies published in English; and (4) first-line or second-line treatment studies for *H. pylori* eradication. Notably, if there were multiple studies of the same study population, the study with the largest sample size was included.

#### Exclusion criteria

We excluded (1) case reports, meeting abstracts, and commentaries; (2) studies with incomplete data; and (3) studies that were duplication or continuation of previous studies.

### Data extraction

Two researchers (J.Y. and Y-M.L.) separately extracted information from all eligible articles and cross-checked each other’s findings. Any inconsistencies in results were resolved by a third senior investigator (X–Y.W.) until a consensus was reached. The following information was extracted for each study: first author, year of publication, country, study design, proportion of patients with peptic ulcers, number of participants, eradication regimens, treatment duration, method for assessing *H. pylori* eradication, interval for assessing *H. pylori* eradication after the therapy, and alcohol consumption. In addition, the quality of each included study was assessed independently by the two researchers using the Newcastle–Ottawa Scale (NOS) on the basis of three broad domains of selection, comparability, and exposure/outcome. Each study was scored on a scale of 0 to 9.

### Statistical analysis

The association between alcohol consumption and the risk of *H. pylori* eradication failure was calculated using ORs and 95% confidence intervals (CIs). The reported OR and 95% CI were used when available and calculated when not available. Heterogeneity between studies was assessed by the Q test and *I*^2^ test. *I*^2^ ≤ 50% and *P* > 0.05 suggested no statistical heterogeneity, and in this case, the fixed-effects model was selected for data analysis; otherwise, the random-effects model was applied. Subgroup analysis was performed to explore the source of heterogeneity. Sensitivity analysis was performed to ensure the stability of meta-analysis results. Funnel plots were used for visual evaluation of publication bias; Egger's and Begg's tests were used for statistical assessment of publication bias. A *P* value of < 0.05 was considered statistically significant. All statistical analyses were performed using Stata software version 15.1 (Stata Corp LLC 4905 Lake Way Drive, College Station, TX 77845 USA).

## Results

### Study identification and selection

**Fig. 1:**
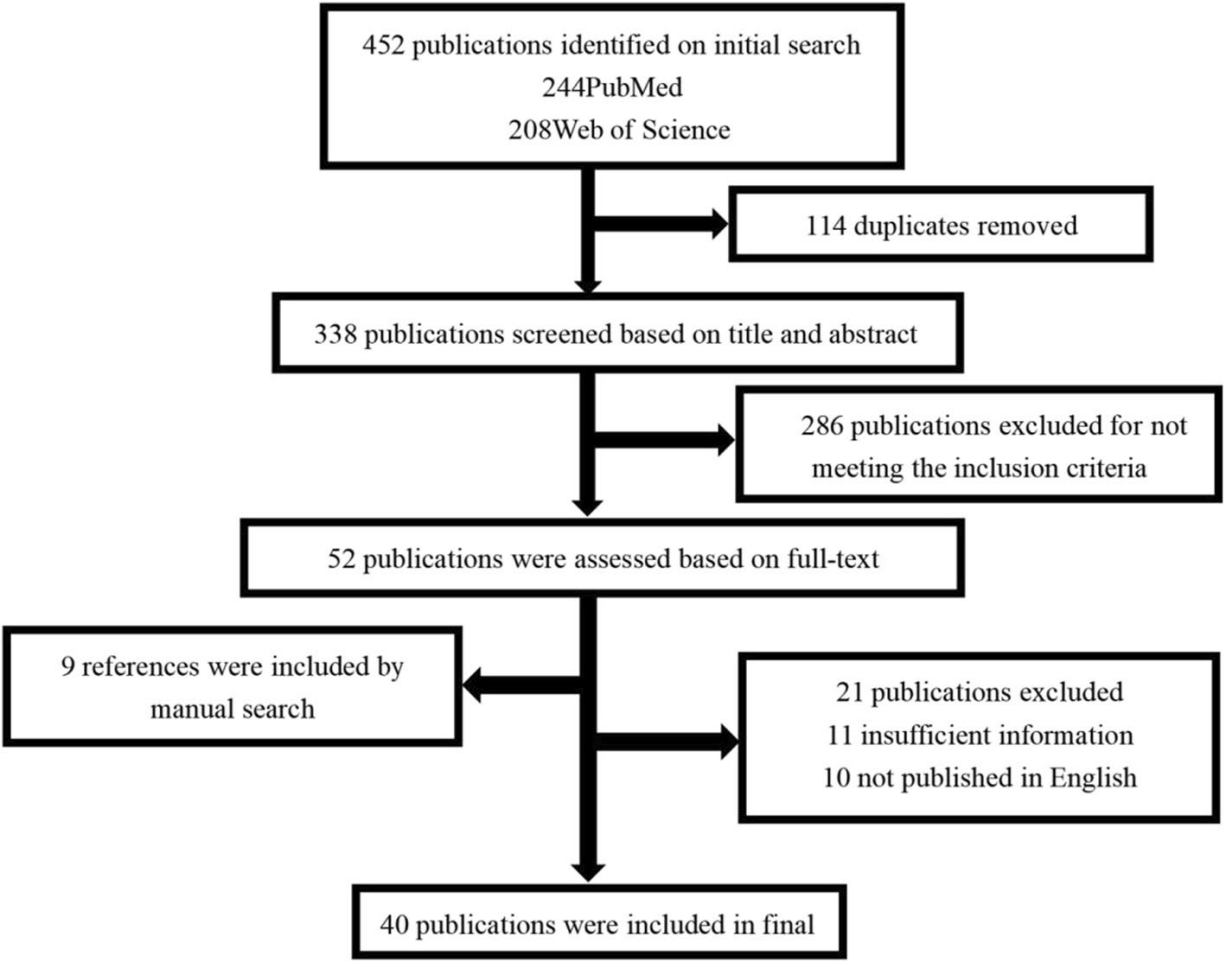
Flow chart of study selection

Figure 1 shows the details of literature retrieval. First, using the abovementioned keywords, 244 and 208 studies were obtained from PubMed and Web of Science, respectively. From among these studies, 114 studies were excluded because of duplication. Then, the titles and abstracts of the remaining 338 publications were reviewed, and 286 studies that did not meet the inclusion criteria were excluded. Then, the full text of the remaining 52 studies was evaluated, and 9 studies were included through manual retrieval of their references. From among these 61 studies, 21 studies were excluded because 11 studies did not contain sufficient data and 10 studies were not published in English. Finally, 40 studies were included in the final analysis.

### Study characteristics

Table 1 shows the characteristics of the 40 studies [1–3, 7, 8, 11–17, 19–46] selected. Among these studies, 24 studies [1–3, 7, 11–13, 16, 17, 22, 30, 32, 34–41, 43–46] were conducted in Asia and 16 outside Asia. Seven studies [15, 23, 26, 30–32, 37] included all patients with peptic ulcers as subjects. Among the studies conducted in Asia, only seven studies [16, 32, 36, 37, 43–45] had data on alcohol intake. Regarding the antibiotic agent(s) administered, clarithromycin was administered in 17 studies, and nitroimidazoles were administered in 3 studies [11, 32, 46]. A VPZ-based therapy regimen was followed in five studies [2, 3, 16, 17, 46]. The NOS results revealed that the average quality score was 6.2 (range, 5–8), indicating that the methodological quality of these studies was generally good.

**Table 1:** Characteristics of included studies

Author (year)	Study design	Country	Men/women	% of patients with peptic ulcer (No.)	Number of participants		Odd ratios (95% CI)	Treatment options	Method for assessing HP eradication	Interval for assessing HP eradication after therapy	Alcohol consumption	NOS
			Age (years)		Drinker	Non–drinker						
Cutler et al. (1993) [14] l	P	US	57/39 54.1 ± 1.5	71 (68/96)	33	63	1.62 (0.54–4.82)	BMT-14d	Endoscopy/UBT	4 weeks	clinic records	7
Labenz et al (1994) [19]	P	Germany	183/222 20–90	94.6 (383/405)	94	311	1.68 (1.01–2.79)	PPI, AMO-7/14d	RUT, histology, UBT	at least 4 weeks	NM	6
Hazell et al. (1997) [20]	P	UK	56/45 21–71	27.7(28/101)	41	60	0.83 (0.25–2.73)	PPI, placebo -14d + PPI, AMO-14d PPI, placebo -14d + PPI, placebo-14d	Endoscopy	4 weeks	NM	5
Olafsson et al. (1999) [21]	P	Norway	108/75 21–85	57 (104/184)	113	52	5.5 (0.69–43.78)	RBC_400_OM-10d RBC_400_SM + 10d RBC_200_OM-10d RBC_200_SM-10d	UBT	8 weeks	4L/year	7
Kamada et al. (1999) [22]	P	Japan	95/42 22–75	58 (79/137)	61	76	1.27 (0.60–2.67)	PCA-7d	UBT	At least 4 weeks	patient interview	7
Fallone et al. (2000) [23]	P	Canada	65/22 16–90	100 (98/98)	45	42	0.57 (0.22–1.48)	BIS, MET, AMO, BIS, MET + 1 placebo, MET + 2 placebos	Gastric biopsies	Every 3 months for 1 year	> 2 g/day	5
Perri et al (2001) [24]	P	Italy	NM 26–75	35 (46/132)	NM	NM	0.94 (0.58–1.53)	PCA-7d	UBT	4–6 weeks	patient interview	5
Maconi et al. (2001) [25]	P	Italy	7/59 47.05/48.05	50 (71/142)	34	95	0.45 (0.12–1.76)	PCA-7d/14d	UBT	1 month + 3 month	outpatient clinic records	7
Silva et al. (2001) [15]	P	Brazil	68/132 16–80	100 (200/200)	29	161	0.66 (0.27–1.58)	PPI, CLA, TIN -7d	Histology/RUT	10–12 weeks	standard questionnaire	6
Mantzaris et al. (2002) [26]	P	Greece	139/138 16–70	100 (149/149)	58	91	1.10 (0.25–4.80)	PAC-10d PBMT-10d	Histology, IMM, CLO	10–12 weeks + 12 month /21–24 months	social alcohol	5
Queiroz et al. (2002) [27]	P	Brazil	39/58 40.5/45.1	60 (58/97)	43	54	0.77 (0.23–2.59)	PPI, CLA, FUR-14d	UBT	3 months	In- and outpatient clinic records	7
Baena et al. (2002) [28]	P	Spain	79/77 47.2 years	56.4 (88/156)	49	107	0.33 (0.13–0.84)	PCA-7d	UBT	4–8 weeks	18.2 g of pure alcohol per day	6
Lin et al. (2002) [43]	P	Taiwan, China	48/30 53.4 years	NM	10	68	1.30 (0.24–6.94)	PBCA-7d	RUT, histology	8 weeks	> 80 g/day	6
Broutet et al. (2003) [29]	RE	France	1726/761 18–87	67 (1838/2751)	782	1364	0.91 (0.75–1.12)	PPI, AMO, ROX-10d/7d PPI, AMO, AZI-10d/7d	UBT	4–6 weeks	Currently	6
Hsu et al. (2005) [44]	P	Taiwan, China	133/67 ≥ 18 years	46 (91/200)	23	177	3.86 (1.40–10.64)	PCA-7d	Endoscopy/UBT	8 weeks	≥ 80 g/day	6
Manes et al. (2005) [8]	P	Italy	171/148 19–79	NM	16	307	1.67 (0.52–5.4)	PPI, CLA, TIN-7d	UBT	4–6 weeks	Outpatient clinic records	5
Ishioka et al. (2007) [30]	RE	Japan	510/91 51.5 ± 9.3 50.7 ± 10.2	100 (601/601)	334	267	0.9 (0.6–1.5)	PCA-7d	UBT	1–2 months	Standard questionnaire	7
Namiot et al. (2008) [31]	P	Poland	155/82 52.2 ± 14.6 47.9 ± 13.9 50.4 ± 15.4	100 (237/237)	103	134	0.48 (0.25–0.93)	OAT/OAC/OCT-7d	Endoscopy	4 weeks	25 g or more alcohol per week	6
Singh et al. (2008) [32]	CC	India	60/7 39.9 + 13.6	100 (67/67)	26	41	2.19 (0.79–6.03)	PPI, AMO, TIN-7d	Endoscopy	4 weeks	More than 30 g in a week	6
Gatta et al. (2010) [33]	P	Italy	110/118 29–53	41.7 (95/228)	175	53	2.01 (0.85–4.70)	PCA-7d	UBT	4 weeks	NM	6
Lee et al. (2014) [45]	P	Korea	1153/1049 52.9 ± 12.8	34.5 (758/2202)	37	2165	0.36 (0.11–1.19)	PCA-7d	UBT	4 weeks	More than 100 g of alcohol a week	5
Lim et al. (2015) [34]	P	Korea	51/47	15.3 (15/98)	17	81	1.33 (0.45–3.86)	PPI, AMO, MOX-7/14d	UBT	4 weeks	NM	5
Kim et al. (2015) [35]	RE	Korea	901/512 14–86	74 (1041/1413)	513	608	1.12 (0.74–1.69)	PCA-7d	UBT/RUT	4 weeks	Clinic records	5
Pan et al. (2015) [11]	P	China	76,485/89787 25–54	NM	8866	34,976	1.16 (1.08–1.23)	PBMT-10d	UBT	45 days	Standard questionnaire	8
Tsai et al. (2016) [36]	RE	Taiwan, China	68/68 57.1 ± 11.3	35.3 (48/136)	90	502	3.786 (1.126–12.690) 0.59 (0.27–1.29)	PCA-7d	Endoscopy/UBT	4-8 weeks	> 80 g/day in men > 40 g/day in women	8
Huh et al. (2016) [37]	RE	Korea	343/171	100 (514/514)	228	286	0.94 (0.59–1.47)	PCA-7-14d	UBT/RUT	At least 4 weeks	≥ 40 g/day in men ≥ 20 g/day in women	7
Noda et al. (2016) [17]	P	Japan	85/61 52–73	14.4 (21/146)	79	62	1.65 (0.53–5.11)	VPZ, AMO, CLA-7d PCA-7d	UBT	1–2 months	Questionnaire	6
Ahn et al. (2017) [38]	P	Korea	34/50 41–73	25 (21/84)	18	66	0.98 (0.19–5.20)	PCA-10d	UBT	6-8 weeks	NM	5
Zhang YW (2018) [12]	RE	China	501/491 46.7 ± 12.4	26.1 (259/992)	231	628	2.52 (1.43–4.41)	FUR, AMO, BIS, PPI—10-14d	UBT or gastric biopsy	> 4 weeks	Clinical Data	5
Chang et al. (2019) [39]	P	Korea	89/101 55.4 ± 10.2	0 (0/190)	65	125	0.53 (0.27–1.06)	PCA-7d	UBT	4 weeks	Collected data	7
Song et al. (2019) [1]	RE	China	273/311	48.8 (285/584)	83	501	0.2 (0.0‐1.8)	PBAF-10d/14d PBTF-10d/14d PBLF -10d/14d	UBT/RUT/histology/FAT	at least 4 weeks	Medical records and telephone interviews	5
Jin et al. (2019) [40]	P	China	128/162 49.32 ± 12.56	0 (0/290)	76	214	1.56 (0.67–3.64)	PBCA-14d	NM	at least 4 weeks	Heavy drinker Social drinker	7
Yi et al. (2019) [13]	P	China	98/87 18–70	16.8 (31/185)	49	136	3.15 (0.59–16.86) 0.99 (0.24–4.07)	PBAF-14d PBCA-14d	NM	4 weeks	Outpatient clinics and inpatient wards	7
Ozeki et al. (2019) [41]	P	Japan	51/158	NM	131	209	0.94 (0.43–2.09) 3.75 (1.57–8.94)	PCA-7d	UBT/Endoscopy + RUT	2 months + 1 year	More than once a week	7
Yao et al. (2019) [7]	RE	Taiwan China	357/362 64.1 ± 9.0 58.3 ± 12.2	19.5 (140/719)	106	613	1.202 (0.663–2.177)	PCA-7d	UBT	6-8 weeks	Medical records	7
Takara et al. (2019)[2]	RE	Japan	261/359 61.03 ± 12.47 62.17 ± 12.01	NM	174	466	1.41 (0.49–4.02) 0.89 (0.55–1.47)	VPZ, AMO, CLA-7d PCA-7d	UBT	at least 5 weeks	Clinic records	7
Okubo et al. (2020) [16]	P	Japan	60/86 22–85	NM	61	85	3.13 (0.98–20.12)	VPZ, AMO, CLA-7d	UBT	at least 4 (4–16 weeks)	> 20 g of pure alcohol daily	6
Eto et al. (2021) [3]	RE	Japan	103/60 20–79	NM	25	138	1.91 (0.63–5.78)	VPZ, AMO-7d	UBT	4 weeks	Clinic records	6
Nkuize et al. (2021) [42]	RE	Belgium	183/279 43.4 ± 10.9 39.1 ± 12.1	NM	114	340	0.570 (0.321–1.011)	PPI, CLA, MET—7-14d; PPI, AMO-5d + PPI CLA, AMO-5d; PBMT-10d; PPI, AMO, CLA, MET-14d;	UBT	At least 6 weeks	Current drinker	7
Kasai et al. (2021) [46]	RE	Japan	NM 34–79	NM	6	27	0.88 (0.08–9.29)	VPA, AMO, MET-7d	UBT	4 weeks	Databases	6

Abbreviations: *P* Prospective study, *CC* Cross-sectional study, *RE* Retrospective study, *HP* Helicobacter pylori, 95% CI 95% confidence interval, *NM* Not mentioned, *US* The United States, *UK* The United Kingdom, *BIS* Bismuth citrate, *AZI* Azithromycin, *PPI* Proton pump inhibitor, *ROX* Roxithromycin, *MET* Metronidazole, *VPZ* Vonoprazan, *AMO* Amoxicillin, *CLA* Clarithromycin, *TIN* Tinidazole, *FUR* Furazolidone, *NIT* Nitroimidazole, *MOX* Moxifloxacin, *PBMT* Proton pump inhibitor, bismuth citrate, metronidazole and tetracycline, *PBCA* Proton pump inhibitor, bismuth citrate clarithromycin, and amoxicillin, *BMT* Bismuth citrate, metronidazole and tetracycline, *OAC* Omeprazole, amoxicillin and clarithromycin, *OAT* Omeprazole, amoxicillin and tetracycline, *OCT* Omeprazole, clarithromycin, and tetracycline, *PCA* Proton pump inhibitor, clarithromycin, and amoxicillin, *PBAF* Proton pump inhibitors, bismuth, amoxicillin, furazolidone, *PBTF* Proton pump inhibitors, bismuth, tetracycline, furazolidone, *PBLF* Proton pump inhibitors, bismuth, levofloxacin, furazolidone, *RBC* Ranitidine bismuth citrate, *OM* Oxytetracycline, metronidazole, *SM* Spiramycin, metronidazole, *UBT* Urea breath test, *RUT* Rapid urease test, *CLO* Campylobacter-like organisms, *FAT* Fecal antigen testing, *IMM* Immunohistochemistry, *NOS* Newcastle Ottawa quality assessment scale

Odds ratios and 95% confidence intervals were used if they were reported in the literature and were calculated if they were not reported

### Association between alcohol consumption and the risk of *H. pylori* eradication failure

**Fig. 2:**
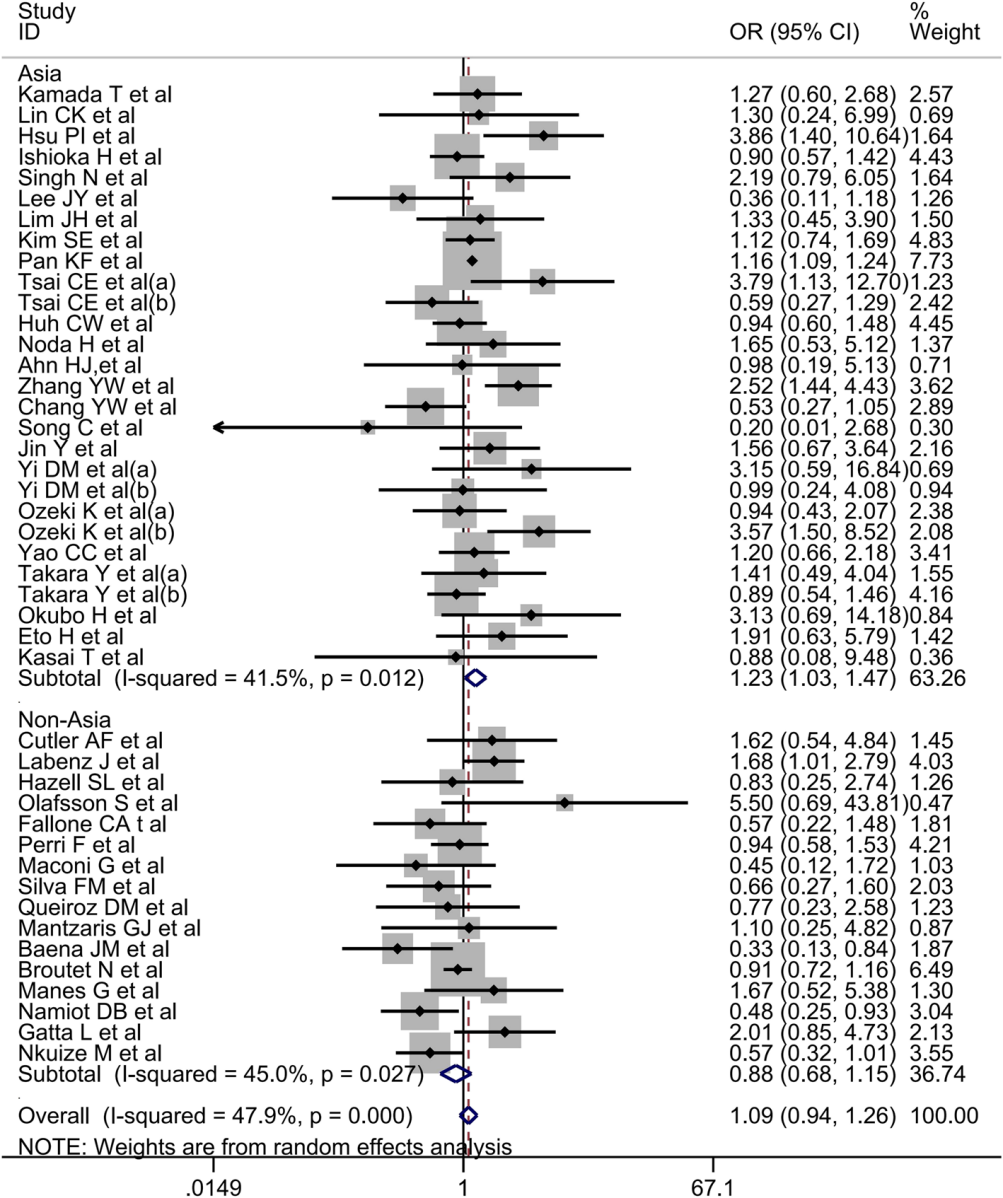
A forest plot of the association between alcohol and *H. pylori* eradication. Subgroup analysis was based on the region

**Fig. 3:**
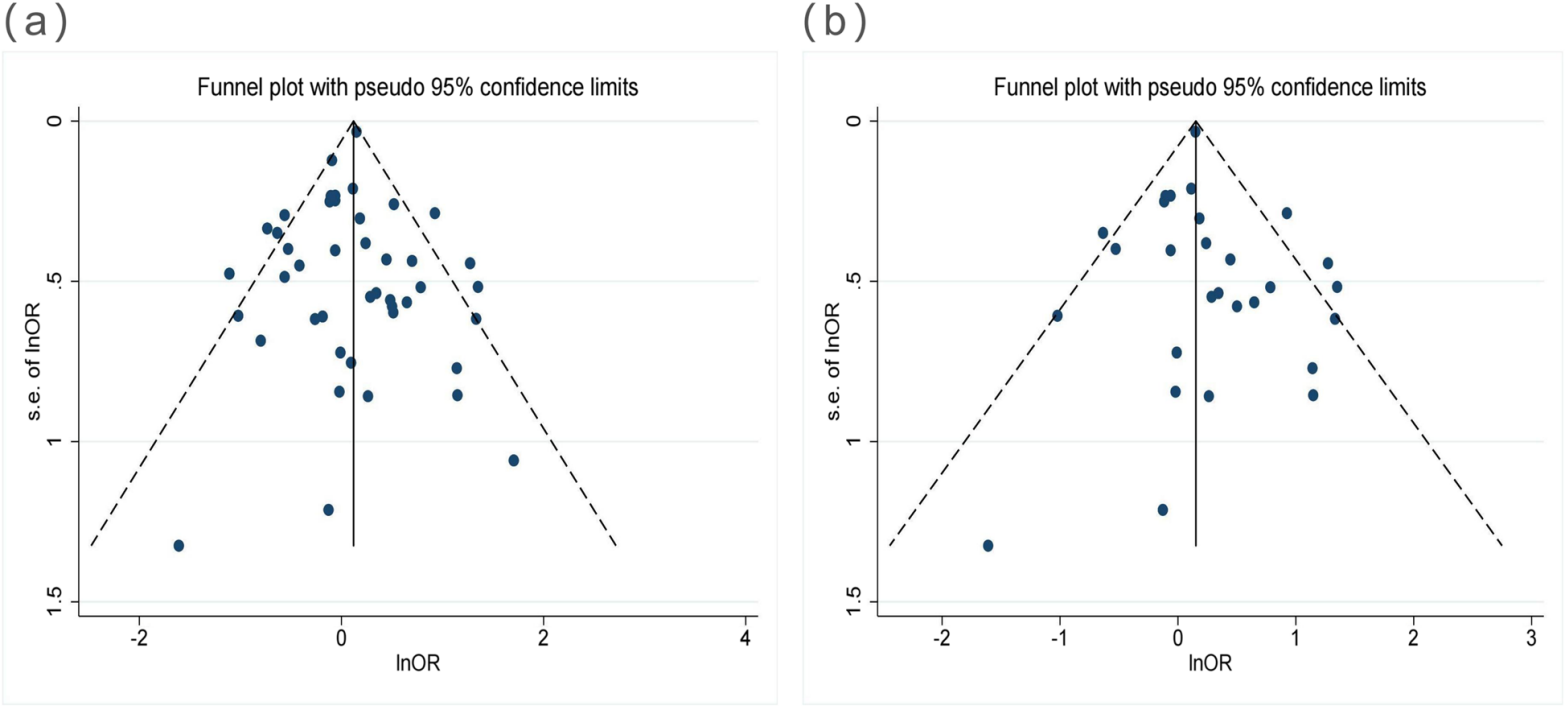
Funnel plots of publication bias (a: All studies included, b: Asian studies). Each round dot represents one study; Begg’s test or Egger’s test also showed no evidence of publication bias in both A and B subfigures

Finally, 40 studies reporting the association between alcohol consumption and *H. pylori* eradication were analyzed. Owing to the significant heterogeneity among these studies (*I*^2^ = 47.9%, *P*-heterogeneity < 0.001), a random-effects model was applied. The risk of *H. pylori* eradication failure was not higher in drinkers than in non-drinkers (OR = 1.09, 95% CI, 0.94–1.26) (Fig. 2). To assess the effect of each individual study on the pooled effect size, sensitivity tests were performed by excluding one study at a time. Withdrawal of any study did not significantly alter the results. The pooled OR ranged from 0.94 to 1.26, confirming the robustness of the results. Neither visual assessment using the funnel plot (Fig. 3a**)** nor statistical assessment with Begg’s test (*P* = 0.401) or Egger’s test (*P* = 0.805) revealed any publication bias.

### Association between alcohol consumption and the risk of *H. pylori* eradication failure in Asia

Herein, 24 Asian studies reported on the effect of alcohol consumption on *H. pylori* eradication. There was significant heterogeneity among these studies (*I*^2^ = 41.5%, *P*-heterogeneity = 0.012), and a random-effects model was applied. The analysis results revealed alcohol as a risk factor for *H. pylori* eradication failure (OR = 1.23, 95% CI, 1.03–1.47) (Fig. 2). Next, sensitivity analysis was performed, and the pooled OR ranged from 1.03 to 1.47, confirming the robustness of the results. The funnel plot (Fig. 3b) was roughly symmetrical, and there was no publication bias on Begg’s test (*P* = 0.489) or Egger’s test (*P* = 0.455).

### Subgroup analysis

The origin of heterogeneity was explored by subgroup analysis (Table 2). No significant differences were observed between the prospective studies (OR = 1.13, 95% CI, 0.92–1.38) and retrospective studies (OR = 1.05, 95% CI, 0.83–1.32), all of which indicated that alcohol consumption did not increase the risk of *H. pylori* eradication failure. When stratified by treatment options, a higher risk of eradication failure was not observed among drinkers undergoing first-line treatment (OR = 1.09, 95% CI, 0.94–1.26) or second-line treatment (OR = 1.25, 95% CI, 0.54–2.93). No significant differences were found between studies having all patients with peptic ulcer (OR = 0.83, 95% CI, 0.65–1.07) and those having a proportion of patients with peptic ulcer (OR = 1.19, 95% CI, 0.93–1.51), all of which indicated that alcohol consumption did not increase the risk of *H. pylori* eradication failure. Urea breath test (OR = 0.98, 95% CI, 0.82–1.16) and other methods (OR = 0.75, 95% CI, 0.51–1.09) used to evaluate *H. pylori* eradication were not associated with a higher risk of *H. pylori* eradication failure among drinkers.

**Table 2:** Subgroup analyses between alcohol consumption and the risk of *H. pylori* eradication failure

Variable		Number of Data	Odd ratios (95% CI), %	*I*^2^ (%)	*P*-value for heterogeneity
Region	Asia	28	1.23 (1.03–1.47)	41.5	0.012
	Non-Asia	16	0.88 (0.68–1.15)	45.0	0.027
Study design	Prospective study	30	1.13 (0.92–1.38)	46.2	0.003
	Retrospective study	14	1.05 (0.83–1.32)	48.8	0.021
Treatment regimen	First-line treatment	41	1.09 (0.94–1.26)	51.4	< 0.001
	Second-line treatment	3	1.25 (0.54–2.93)	0.00	0.952
Proportion of subjects with peptic ulcer	All	7	0.83 (0.65–1.07)	20.8	0.271
	Portion	24	1.19 (0.93–1.51)	50.9	0.002
Method for assessing HP eradication	Urea breath test	23	0.98 (0.82–1.16)	39.7	0.027
	Others	7	0.75 (0.51–1.09)	16.0	0.308

Abbreviations: *HP* Helicobacter pylori, *VPZ* Vonoprazan

Moreover, in Asian patients (Table 3), a treatment duration of > 7 days was associated with higher risk of *H. pylori* eradication failure (OR = 1.17, 95% CI, 1.10–1.25). Alcohol consumption was found to increase the risk of *H. pylori* eradication failure when the treatment regimen did not include clarithromycin (OR = 1.18, 95% CI, 1.10–1.26) or when the treatment regimen included nitroimidazoles (OR = 1.16, 95% CI, 1.09–1.24). Notably, alcohol consumption also decreased the eradication rate when bismuth-containing quadruple therapy (OR = 1.17, 95% CI, 1.10–1.25) was used to treat *H. pylori* infection in Asian patients; however, the *H. pylori* eradication efficacy of the VPZ-based therapy regimen (OR = 1.73, 95% CI, 0.98–3.05) remained unaffected by alcohol consumption. In addition, seven of the Asian studies provided information on the amount of alcohol intake. In these studies, increase in alcohol intake (> 40 g/day) was associated with a higher risk of *H*. *pylori* eradication failure (OR = 3.17, 95% CI, 1.56–6.41).

**Table 3:** Subgroup analyses between alcohol consumption and the risk of *H. pylori* eradication failure in Asia

Variable		Number of Data	Odd ratios (95% CI), %	*I*^2^ (%)	*P*-value for heterogeneity
Duration of therapy	7 days	19	1.23 (0.94–1.60)	47.4	0.012
	> 7 days	8	1.17 (1.01–1.25)	40.4	0.109
Antibiotic types	Clarithromycin	20	1.15 (0.91–1.45)	41.9	0.026
	Non-Clarithromycin	8	1.18 (1.10–1.26)	44.5	0.082
	Nitroimidazoles	3	1.16 (1.09–1.24)	0.00	0.461
	Non- Nitroimidazoles	25	1.24 (0.99–1.57)	46.2	0.007
Treatment options	VPZ-based therapy	5	1.73 (0.98–3.05)	0.00	0.897
	bismuth-containing quadruple therapy	7	1.17 (1.10–1.25)	40.7	0.028
Alcohol consumption	< 40 g/day	4	1.15 (0.54–2.45)	59.1	0.062
	> 40 g/day	3	3.17 (1.56–6.41)	0.00	0.520

Abbreviations: *HP* Helicobacter pylori, *VPZ* Vonoprazan

## Discussion

### Main findings

Our meta-analysis included 40 studies and assessed the potential association between alcohol consumption and the risk of *H. pylori* eradication failure. The overall pooled OR of drinkers versus non-drinkers was 1.09 (95% CI: 0.94–1.26), suggesting that alcohol consumption was not associated with and did not exacerbate the risk of *H. pylori* eradication failure. Stratified by the study region, our meta-analysis showed that increased alcohol consumption was associated with increased risk of *H. pylori* eradication failure in Asian studies. Compared to controls, individuals with alcohol consumption of over 40 g/day were more than three times likely to encounter *H. pylori* eradication failure. The association between alcohol consumption and the risk of *H. pylori* eradication failure is supported by several biological mechanisms. First, alcohol consumption activates gastric acid secretion, leading to a decrease in stomach pH, which promotes the breakdown of antibiotics and reduces their effectiveness [41]. In addition, ethanol can reportedly significantly affect the absorption rate of amoxicillin [47]. Second, alcohol has a strong induction effect on the liver enzyme CYP2C19 [48]. Meanwhile, for the CYP2C19 genotype, *H. pylori* eradication rates were reported to be lower in the rapid and intermediate metabolizer groups of proton pump inhibitors (PPIs) than in poor metabolizer groups [10]. Therefore, alcohol may affect the eradication rate by inducing CYP2C19. Moreover, alcohol can also alter the gastric microenvironment, thus affecting the stability of antibiotics and leading to a decrease in the eradication rate [44].

The discrepancy between the results of Asian and non-Asian studies may be explained by potential differences in their regional populations, such as genetic and physical differences. There are two major aldehyde dehydrogenase (ALDH) isoenzymes in the liver: cytoplasmic ALDH1 and mitochondrial ALDH2. The primary pathway by which ethanol is metabolized involves its degradation by alcohol dehydrogenase into acetaldehyde (an intermediate metabolite), which is then acted upon by ALDH and converted into acetic acid [49]. Previous studies have reported a widespread prevalence of ALDH deficiency in Oriental populations [50], and thus, it can be interpreted that Asians metabolize ethanol less efficiently than non-Asians. In addition, for the same BMI, Asians have a higher body fat percentage than Caucasians [51], and alcohol is not easily absorbed by adipose tissues because of its low fat solubility [52]. These factors also contribute to higher plasma alcohol concentrations in Asian populations than in non-Asian populations. These results suggest that alcohol consumption impacts Asian populations more strongly and therefore influences *H. pylori* eradication to a greater extent.

In Japan, the standard treatment for *H. pylori* eradication is a seven-day triple therapy, namely PPI or P-CAB combined with amoxicillin, clarithromycin, or metronidazole [53]. As a novel P-CAB, VPZ has been approved for *H. pylori* eradication in recent years [16]. Our findings showed that alcohol consumption did not detrimentally affect the efficacy of the VPZ-based therapy regimen, and although the reasons for this remain unknown, we can speculate that the strong inhibition of gastric acid by VPZ conceals the effect of alcohol on the *H. pylori* eradication rate [2]. Therefore, to further explore this phenomenon, prospective studies on the efficacy and safety of the VPZ-based therapy regimen in a larger population are warranted, particularly in other regions besides Japan. Future research should also focus on exploring the mechanism by which the VPZ-based therapy regimen remains unaffected by alcohol intake.

### Strengths and limitations of analysis

The present study has several limitations. First, several confounding factors differed among studies, such as BMI, race, study design, and treatment regimen; this may have influenced the results of our study. Second, studies have shown that the existence of the dose–response gradient can improve the quality of evidence [54]. Since only seven Asian studies provided information on alcohol intake, the questionnaire was not an ideal source of data in most Asian studies included herein. Therefore, we could only incorporate these seven studies into the dose–response analysis of alcohol consumption and *H. pylori* eradication. Further studies are needed to examine the relationship between different alcohol intake doses and the corresponding risk of *H. pylori* eradication failure. Third, we did not analyze the effect of the type of alcohol consumed on *H. pylori* eradication rates, and since different types of alcohol have different potentially relevant properties in this regard, for example, some studies have shown that wine has antibacterial properties, this perspective deserves further exploration [55, 56]. Finally, most of the included studies did not test for antibiotic resistance in their trials, which may have influenced the *H. pylori* eradication rate. Nonetheless, despite these limitations, to our knowledge, this is the first meta-analysis to explore the relationship between alcohol consumption and the risk of *H. pylori* eradication failure. In addition, VPZ is a novel P-CAB that has been recently approved in Japan for *H. pylori* eradication. We included relevant literature to provide the first comprehensive analysis of whether alcohol consumption has an effect on *H. pylori* eradication in individuals undergoing the VPZ-based therapy. Further large-scale, multicenter prospective studies are warranted to verify our results.

## Conclusion

In summary, our meta-analysis suggests that alcohol consumption increases the *H. pylori* eradication failure rate in Asian populations. Alcohol intake of > 40 g/day was associated with *H*. *pylori* eradication failure. It should also be noted that alcohol consumption may not negatively affect *H. pylori* eradication rates when the VPZ-based therapy regimen is being used to treat *H. pylori* infection in Asian populations. Therefore, we suggest that for Asian populations, drinkers should abstain from alcohol to improve the *H. pylori* eradication rate; furthermore, in patients having difficulty in abstaining from alcohol, the VPZ-based therapy regimen can be adopted to avoid the influence of alcohol on treatment efficacy.

## Data Availability

The datasets used and/or analyzed during the current study are available from the corresponding author on reasonable request.
